# The Impact of School-Based Nutrition Interventions on Parents and Other Family Members: A Systematic Literature Review

**DOI:** 10.3390/nu14122399

**Published:** 2022-06-09

**Authors:** Eman Abderbwih, Melani Ratih Mahanani, Andreas Deckert, Khatia Antia, Nisreen Agbaria, Peter Dambach, Stefan Kohler, Olaf Horstick, Volker Winkler, Amanda S. Wendt

**Affiliations:** 1 Heidelberg Institute of Global Health, Heidelberg University Hospital, 69120 Heidelberg, Germany; melani.mahanani@uni-heidelberg.de (M.R.M.); a.deckert@uni-heidelberg.de (A.D.); khatia.antia@uni-heidelberg.de (K.A.); nisreen.agbaria@uni-heidelberg.de (N.A.); peter.dambach@uni-heidelberg.de (P.D.); stefan.kohler@uni-heidelberg.de (S.K.); olaf.horstick@uni-heidelberg.de (O.H.); volker.winkler@uni-heidelberg.de (V.W.); 2 Potsdam Institute for Climate Impact Research, Member of the Leibniz Association, P.O. Box 601203, 14412 Potsdam, Germany; wendt@pik-postdam.de

**Keywords:** school-based, nutrition intervention, parent, family, systematic review

## Abstract

Little is known about the impact of school-based nutrition interventions on parents and other family members. This systematic review aims to explore the impact of school-based nutrition interventions on different parental/family outcomes, mainly dietary intake, nutrition knowledge, and health outcomes. PubMed, Web of Science, PsycINFO, EconLit, Cochrane Reviews, and Google Scholar were systematically searched for controlled trials or natural experiments measuring the impact of school-based nutrition interventions, with or without parental involvement, on parents/families of school children. Twenty-two studies met the inclusion criteria. Of which, 15 studies assessed the impact of school-based nutrition interventions on parental/family dietary intake, 10 on parental/family nutrition knowledge, and 2 on parental/family health outcomes. Inconsistent results were found for parental dietary intake with six studies reporting favorable effects. Most studies found improved parental nutrition knowledge. Positive impacts were seen by both studies that assessed the impact on a parental health outcome. Overall, we found that there is potential for school-based nutrition interventions to result in positive effects for parents, in particular for nutrition knowledge. More research is needed to assess the impacts of school-based nutrition interventions on parents and other family members and to assess important intervention characteristics in creating a positive impact.

## 1. Introduction

The prevalence of non-communicable diseases (NCDs) such as diabetes, cardiovascular diseases, and cancer is continuing to increase worldwide. NCDs already account for 41 million deaths annually, representing 71% of the global deaths [1]. According to the World Health Organization (WHO), NCDs can be controlled by reducing associated risk factors including tobacco use, excessive alcohol use, physical inactivity, and unhealthy diet [1].

The role of diet in the development and control of NCDs is well established [2,3]. Dietary risk factors including low intake of whole grains, fruits, and vegetables as well as high intake of sodium, trans fats, and sugar-sweetened beverages (SSBs) were estimated to account for 11 million deaths and 255 million disability-adjusted life years (DALYs) in 2017—with high sodium and low fruit and whole grain consumption as the leading dietary risk factors [2]. Therefore, interventions aiming to promote healthy diets are essential for the prevention and control of NCDs and their associated morbidities and mortality [4].

The exposure to many NCD risk factors starts often early in life, with severe consequences in later life [5,6]. For example, childhood overweight and obesity were found to be associated with a higher risk of premature mortality and physical morbidity during adulthood [7]. It is therefore important that preventive health interventions target children and adolescents [8]. Schools are one of the key settings through which nutrition and other health interventions can be delivered. This setting has been recognized as an efficient and effective channel for reaching a wider population [9]. There have been several meta-analyses and systematic reviews exploring the effectiveness of school-based health interventions among children and adolescents. Some reviews have demonstrated promising results on children’s and adolescents’ anthropometry [10,11,12], dietary intake [10], and physical activity [12]. In addition to children, school-based health interventions may also have impacts on parents. For example, a systematic review on school-based stroke education suggested that children might be able to convey information about strokes to their parents [13]. Another study reported evidence of a positive effect from health education at primary school on parents engaging in light physical activity [14].

A large segment of the population, including parents and other family members, can benefit from interventions conducted in a school setting. However, the impact of school-based nutrition interventions on parents has not been systematically reviewed. Given the potential effect of these interventions on parents, we sought to conduct a systematic review to explore whether school-based nutrition interventions on school children, with or without parental involvement, have an impact on dietary intake, nutrition knowledge, and health outcomes of their parents and/or other household family members.

## 2. Materials and Methods

This systematic literature review followed the reporting guidelines of Preferred Reporting Items for Systematic Reviews and Meta-Analyses (PRISMA) (see Table S3) [15]. This review is not registered in any protocol registry.

### 2.1. Search Strategy and Eligibility Criteria

A systematic literature search was performed in the following databases: PubMed, Web of Science, PsycINFO, EconLit, Google Scholar, and Cochrane Reviews. The search strategy was developed based on the following three categories: (i) setting, (ii) intervention, and (iii) target population. It was then adapted according to the different structures of the databases (see Table S1 for the search strategy of each database). No date or language restrictions were applied. Additionally, the reference list of each included article was screened for potentially eligible studies. We searched Google Scholar for grey literature by screening stepwise packages of 50 hits until a package did not contain relevant results. All searches were last updated in June 2021.

Each study had to meet the following criteria to be included in this review: (1) be a controlled trial, with or without random assignment, or natural experiment; (2) target school-age children and/or adolescents (5–18 years), with or without parental involvement; (3) evaluate school-based interventions with nutrition as the primary component; and (4) report the impact of interventions on at least one of the following parental/family outcomes: (a) dietary intake, (b) nutrition knowledge, or (c) health outcome. Studies targeting parents only, preschoolers, or children with specific health issues (e.g., overweight or obesity) were excluded. We also excluded interventions related to eating disorders and oral health.

### 2.2. Study Selection and Data Extraction

All studies retrieved from the literature search were imported into EndNote X9 software to eliminate duplicates. The remaining unique studies were then imported to Rayyan QCRI [16]. Two reviewers (E.A. and M.R.M.) independently screened the titles and abstracts. Afterwards, the full texts of the remaining articles were screened for eligibility by the same reviewers. Any uncertainty in the decisions was addressed by a second opinion from a member of the author team (V.W. and A.S.W.). When the full texts of potentially eligible articles were not available, the authors were contacted. For the data extraction, a pre-designed and piloted extraction form was used. This form included information on the following: authors, country, study design, study participants, intervention components, theoretical framework, intervention duration, outcome measures, and findings.

### 2.3. Quality Assessment

The quality assessment was done based on parental data. Two reviewers (E.A. and M.R.M.) independently assessed the quality of included studies using the Quality Assessment Tool for Quantitative Studies [17]. This tool was selected for this review because it can be used to assess the methodological quality of a wide range of study designs in public health. It assesses the quality of studies based on the following components: selection bias, study design, confounders, blinding, data collection methods, and withdrawals and dropouts. Each of these components was rated as “strong”, “moderate”, or “weak” based on predefined criteria as described in the tool’s dictionary. The overall quality rating of each study was then determined based on the ratings of the above six components as “strong” (no component with a weak rating), “moderate” (one component with a weak rating), or “weak” (two or more components with weak ratings). The discrepancies between reviewers were resolved through discussion until consensus was reached.

## 3. Results

### 3.1. Literature Search Results

**Figure 1:**
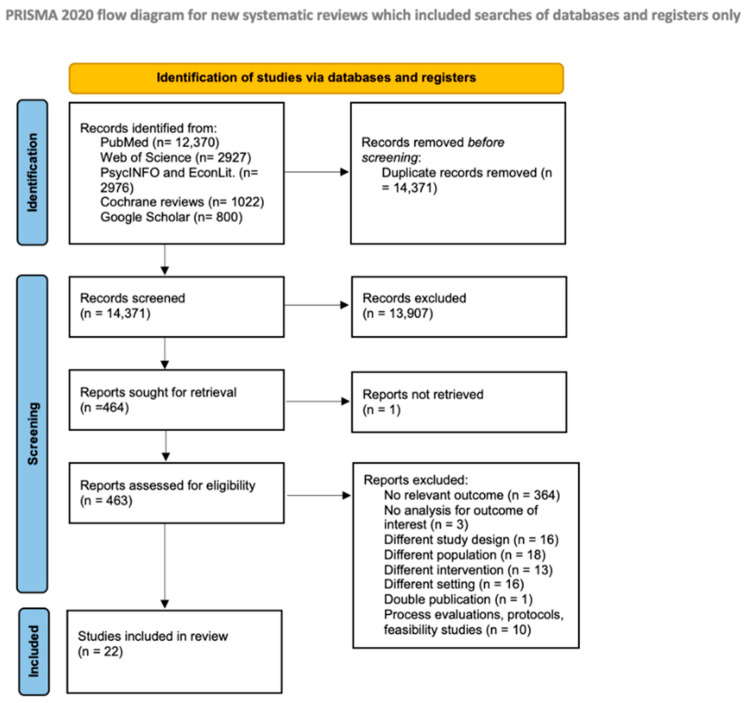
PRISMA flow diagram of study selection.

Our literature search retrieved 20,095 articles. After removal of duplicates, a total of 14,371 articles remained for the title and abstract screening, which yielded 464 articles for the full text assessment. Finally, 22 articles met our inclusion criteria and were thus included in our review. Only one non-English study (Chinese) met the inclusion criteria and was included in our review [18]. Two of the included studies had the same sample, but different outcomes were reported [19,20]. Additionally, one study [21] reported the long-term impact of another included study [22]. Figure 1 shows the results of the search strategy and reasons for exclusion.

### 3.2. Study Characteristics

The study characteristics and quality rating of the included studies are presented in Table 1.

### 3.3. Countries and Study Design

Of the 22 included studies, seven were conducted in the United States [24,25,26,29,33,34,35], five in China [18,19,20,36,38], and five in Europe (two in Norway [23,32], two in the Netherlands [21,22] and one study included samples from Spain, Norway, and the Netherlands [37]). The remaining studies were conducted in the following countries: Kenya [27], Sri Lanka [28], Jamaica [30], Australia [31], and the UK [39]. Regarding study designs, ten of the included studies were RCTs [18,20,23,27,28,29,33,34,37,38], ten were non-RCTs, of which nine utilized pre-post-test study designs [21,22,24,25,26,30,35,36,39], and one used a post-test study design [31]. Finally, one study had a natural experimental study design [32].

### 3.4. Participant Characteristics

All of the included studies recruited students from first through eighth grade. One study included a mixed sample of kindergarten and primary school students [24]. Most of the studies (*n* = 15) included both parents. Three studies included only mothers [28,30,37] and two studies included one adult family member [19,20]. Finally, one study included parents and siblings [27]. The sample size of parents/families varied largely, ranging from 47 [39] to 1698 participants [34].

### 3.5. Intervention Characteristics

Most of the interventions (*n* = 16) had multiple components and involved more than one domain, including the classroom, school-wide, family, and community outreach. The remaining interventions were based on a single component, including the daily distribution of snacks [27], free or subsidized fruits and vegetables [32], and providing lessons and/or training for school children [28,30].

Most of the interventions (*n* = 15) included parent/family components. Different approaches were used to involve parents, including sending messages to parents through mail, newsletters, text-messages, pamphlets, handouts, and/or other educational materials; collaborative homework and activities for children to complete with their parents; and parent meetings, events, lectures, and workshops.

The intervention duration varied greatly, ranging from five weeks [26] to two years [27,31]. Only six interventions were guided by theoretical frameworks, including Social Cognitive Theory (SCT) [25,34,35], Social Learning Theory (SLT) [26,33], Theory of Planned Behavior (TPB) [35], and the Social-Ecological Model [29].

### 3.6. Quality Assessment

Eleven of the included studies had an overall “weak” quality rating [18,21,22,23,24,27,29,30,31,36,39], five had a “moderate” quality rating [25,32,33,35,37], and six had a “strong” quality rating [19,20,26,28,34,38]. Table S2 summarizes the quality assessment for each included study. For the selection bias component, five studies were rated as “weak” [24,25,31,35,39], mainly due to the lack of representativeness of samples to the target population, and low or unreported participation rate. Four studies did not explicitly report the study design and thus were rated as “weak” [24,30,36,39]. One study had a natural experiment design and thus was rated as “moderate” [32]. Regarding the confounder component, six studies were rated as “weak” [18,24,29,31,36,39] mainly due to a lack of reporting about controlling of potential confounders. With respect to blinding, one study was not blinded to the outcome assessors nor to the study participants and thus was rated as “weak” [27]. The remaining studies were rated as “moderate” primarily due to a lack of reporting about the blinding status of outcome assessors and/or study participants. For the data collection method component, ten studies were rated as “weak” mainly due to a lack of reporting about the validity and reliability of the data collection measures. Finally, nine studies were rated as “weak” for the withdrawal and dropout component, due to a high [21,22,23,24,31,37] or unreported attrition rate [29,36,39].

### 3.7. Parental/Family Outcomes

The impact of school-based nutrition interventions on parental/family outcomes along with the outcome measures are presented in Table 2. Additionally, Table S4 and Figure S1 in the Supplementary Materials present a summary of the findings.

#### 3.7.1. Dietary Intake

Mixed results were seen among the 15 studies that evaluated the impact of interventions on parental dietary intake. Of these 15 studies, 7 found no significant intervention effects on dietary intake outcomes [23,26,28,29,33,37,38]. One other study reported a general decline in dietary quantity and quality among all study groups of parents of children receiving supplementary snacks at schools and in the control group. The decline in food quantity was only significant for the parents of the vegetarian group. Significant declines in at least one of the markers of dietary quality were also seen among parents of the vegetarian, meat, and control groups [27]. Nevertheless, as reported in the study, this decline could be attributed to crop failure caused by a failed rainy season. In this study the impact of the intervention was also assessed for siblings of school children. However, we did not consider the results on siblings since it was combined with the results of school children.

The remaining seven studies reported significant intervention effects in the intervention groups. Of these, four measured parents’ change in fruit and vegetable intake. One study found a significant increase in the consumption of fruits, vegetables, and fruits and vegetables combined at the first follow-up (8 weeks post-baseline) compared to the baseline and control group. However, post-intervention, a significant increase was only found for fruit consumption in the intervention group [35]. The second study found a significant difference in vegetable consumption but not for fruits or fruits and vegetables combined immediately post-intervention. No significant differences were found for parental fruit and vegetable consumption at 1-year post intervention [34]. The third study reported a significant increase in fruit and vegetable consumption by parents in the intervention group at the first follow-up (15 months post-baseline). At 20 months post-baseline follow-up, the average fruits and vegetables consumed by parents/guardians of the pilot schools was not significantly different from either the baseline or the comparison school [39]. Finally, one study found that the parents of children who received the intervention had a significantly higher consumption of fruits than parents of children without the intervention. No significant differences in vegetable intake between groups were seen [32].

For the three remaining studies that assessed the dietary intake outcome, one study (two papers) reported a significant reduction in salt intake among family members of the intervention group compared to those of the control group [20], which led to a decrease in iodine consumption [19]. However, the iodine intake remained adequate despite this decrease in salt intake.

Finally, one study reported a significant reduction in parents’ self-reported intake of refined grains, but no significant differences were observed between groups for whole grain intake [25].

Most of the studies collected data via self-reporting, including Food Frequency Questionnaires (FFQs), 24-h recall, and questionnaire items, to assess dietary intake. The recall periods of FFQs varied between studies and were not reported in all studies. Biological markers were used to measure food intake in two studies. In these studies, 24-h urinary sodium [20] and iodine [19] excretion were used to measure salt and iodine intake.

Only three studies reported the guidelines used to estimate the serving size of food items, including the 2005 Dietary Guidelines for Americans [25,40], National Cancer Institute (5 a day guideline) [34,41], and USDA 2010 Dietary Guidelines [35,42].

#### 3.7.2. Nutrition Knowledge

Ten studies assessed the impacts of interventions on nutrition knowledge of parents [18,21,22,24,26,30,31,34,36,38], of which seven reported significant positive intervention effects. For the remaining three studies, one found a positive effect on the knowledge of five a day serving but not on low-fat preparation knowledge at the first follow-up. No significant intervention effect on nutrition knowledge was observed at the second follow-up [34]. Finally, one study (two papers) found no significant differences between the intervention and control groups at the first [22] and second follow-ups [21].

All ten studies used questionnaires to measure nutrition knowledge. Seven of the questionnaires were self-administered [18,21,22,26,31,34,38] and two were interviewer-administered [24,30]. One study did not report the questionnaire’s mode of delivery [36].

#### 3.7.3. Health Outcomes

Only two studies assessed the impacts of nutrition interventions on parental/family health. One study observed a significant reduction in weight and body mass index among intervention group mothers as opposed to the controls [28]. In this study, weight and height were measured at baseline and immediately post-intervention by trained study team members. The second study evaluated the differences between intervention and control groups in blood pressure using a validated automatic blood pressure monitor by trained researchers [20]. At the end of the intervention, both groups showed an increase in the systolic and diastolic blood pressure as compared to baseline. Nevertheless, the increase in systolic blood pressure was smaller in the intervention group than that observed among the control group. The mean effect on systolic blood pressure for the intervention group versus the control group was −2.3 mm Hg (95% confidence interval −4.5 to −0.04 mm Hg). No significant differences were observed in diastolic blood pressure between the groups.

## 4. Discussion

This systematic review sought to explore the impact of school-based nutrition interventions on three parental/family outcomes: dietary intake, nutrition knowledge, and health outcomes.

For the dietary intake, mixed results were reported across studies, with only 42% of the studies reporting positive impacts. A previous systematic review found that school-based nutrition interventions with durations between six weeks and five months were most effective in modifying the dietary behaviors of pre-adolescents and adolescents. It was also found that interventions with durations that are shorter than six weeks or longer than five months were less likely to result in positive effects [43]. Here, we observed a similar trend. Most of the interventions that were ineffective in changing parental dietary intake had a duration longer than five months. We did not find differences between effective and ineffective interventions in terms of intervention components (i.e., single component, multi-component) and parental involvement.

Most of the included studies that assessed the impact of interventions on dietary intake have focused on fruit and vegetable intake, either combined or separately. In our systematic review, the evidence on whether interventions were more effective for fruit or vegetable intake was equivocal. In contrast, a previous systematic review and meta-analysis found that school-based nutrition interventions were effective in improving the fruit intake of children with minimal impact on vegetable intake [44].

Due to the limited number of included studies that assessed the long-term impact of school-based nutrition interventions on the dietary intake of the parents, we were unable to determine the sustainability of the impacts of these interventions over time. Similarly, a previous systematic review reported a lack of evidence on the long-term impact of interventions to increase vegetable intake among children [45].

With regard to nutrition knowledge, most studies reported a statistically significant favourable impact from school-based nutrition interventions on parental nutritional knowledge. Different aspects of nutrition knowledge were studied, such as knowledge about daily recommendations for fruits, vegetables, fat, and salt intake; knowledge about diet and its relationship to diseases; and general nutrition knowledge. However, different nutrition knowledge assessment tools were used across studies. Additionally, some of these assessment methods were not validated. This may limit the comparability between studies. However, results suggest an improved nutrition knowledge among parents as a result of school-based interventions.

Only two studies included in this review assessed the impact of a nutrition intervention on parental health outcomes. These two studies had a strong methodological quality and demonstrated some promising results. Gunawardena et al. [28] reported a significant reduction in the weight of mothers of the intervention group children as compared to the control group. He et al. [20] reported that the family of the intervention group had a significantly smaller increase in systolic blood pressure compared to the control group. The small number of studies makes it difficult to draw a conclusion about this outcome. Interestingly, although these two studies had no direct parental involvement, they demonstrated positive results.

Previous systematic reviews were inconclusive about whether interventions with parental involvement improved child outcomes. One systematic review suggested that the higher involvement of parents in school-based nutrition interventions resulted in better dietary changes among children in comparison with interventions with lower parental involvement [46]. However, interventions without parental involvement were effective in improving dietary intake when they included repeated fruit and vegetable exposure. Another review reported inconsistent evidence on the effect of parental involvement on child health behaviors [47]. The differences in results between the two reviews could be explained by the different natures of the studies included in each review. The first review compared studies with high parental involvement to studies with low or no parental involvement. The second review, however, only included studies that compared the outcomes of the same intervention with and without parental involvement. Additionally, only a limited number of studies were included in the second review. Given the above evidence, parental involvement may influence the impact of school interventions on parents and/or other family members. However, there was insufficient evidence to assess this association in our review. More comprehensive studies are needed to assess the impact of parental involvement in school-based nutrition interventions on parents themselves or other family members. It is noteworthy that only two included studies assessed the impact of school-based nutrition interventions on family members other than parents.

The findings of this review emphasize the potential for school-based interventions in improving certain parental/family outcomes (i.e., parental nutrition knowledge). The findings also show a lack of systematic assessment of the effects of school-based interventions on the family, and it highlights to public health professionals that more research is needed to explore the potential impacts of school-based nutrition interventions beyond children.

Our systematic review has several strengths. First, to our knowledge, this is the first systematic review on the impact of school-based nutrition interventions on parents and other household family members. Second, despite the importance of studying the impact of school-based nutrition interventions on parents and other family members, it is often reported as a secondary outcome. For this reason, we used a broad search strategy that yielded a large number of studies that were screened for eligibility. Additionally, our search was not restricted by date, language, or geographical location. This systematic review has limitations as well. The high variability between studies, especially in terms of intervention components, assessment tools, intervention durations, and follow-up periods, made it difficult to compare results across studies. Furthermore, only six of the included studies had a strong quality rating. The remaining studies had moderate to weak ratings.

## 5. Conclusions

Overall, we found that there is potential for school-based nutrition interventions to result in positive effects for parents, in particular for nutrition knowledge, with less evidence for dietary intake or health outcome impacts. More research is needed to assess the impacts of school-based nutrition interventions on parents and other family members and to assess which intervention characteristics are important for positive impacts.

## Figures and Tables

**Table 1:** General characteristics and quality ratings of included studies.

Authors Year, Country	Design	Participants	Intervention	Theoretical Framework	Intervention Duration	Intervention Components	Parental/ Family Outcomes of Interest	Quality Rating
Bjelland et al., 2015 [23], Norway	CRCT	Sixth-grade students: *n* = 1418; Mothers: *n* = 849; Fathers: *n* = 680	Health In Adolescents	NR	20 mo.	Classroom components, home/parents’ components, school wide components, and leisure time activities	Parental intakes of sugar-sweetened soft drinks and sugar- sweetened fruit drinks; Intake of FV	Weak
Blom-Hoffman et al., 2008 [24], USA	Non-RCT	Kindergarten and first-grade students: *n* = 297; Parents: *n* = 80	Fruit and Vegetable Promotion Program	NR	16 mo.	Morning announcements highlighting the FV of the day and an associated fact; posters; Dole CD-ROM during computer special or in the classroom; and assignments and take-home books	Parental knowledge of the “5 a Day” message	Weak
Burgess-Champoux et al., 2008 [25], USA	Non-RCT	Fourth- and fifth-grade students/ parent pairs: I: *n* = 67, C: *n* = 83	The ‘Power of 3: Get Healthy with Whole Grain Foods’	SCT	5 mo.	Five-lesson classroom curriculum, school cafeteria menu modifications, and family involvement	Parental self-reported intake of refined- grain/whole grain foods	Moderate
Crockett et al., 1989 [26], USA	Non-RCT	Parents of third-grade students: *n* = 465	Hearty Heart and Friends & Hearty Heart Home Team	SLT	5 wk.	Hearty Heart and Friends: a third-grade curriculum; Hearty Heart Home Team: a five-week parent-taught intervention that is mailed to students’ homes; Hearty Heart and Home Team: both school and parent-taught; and Control group: no intervention	Parental knowledge about diet and its relationship to CVD and dietary intake	Strong
Dong et al., 2019 [18], China	CRCT	Fourth-grade students: I: *n* = 1361, C: *n* = 1364; Parents: I: *n* = 1306, C: *n* = 1340 C	Salt Reduction Model	NR	8 mo.	Educational materials for salt reduction; watching popular science movies and children’s animations; six monthly salt reduction training sessions for students; publicity boards, campus radio, newspapers, etc. for publicity; winter vacation activities; and salt reduction theme activity and family component.	Parental knowledge of salt intake	Weak
Gewa et al., 2013 [27], Kenya	CRCT	First-grade students and siblings: vegetarian group: *n* = 80, meat group: *n* = 96, milk group: *n* = 101, control: *n* = 63; Parents: vegetarian group: *n* = 79, meat group: *n* = 91, milk group: *n* = 100, control: *n* = 72	The Child Nutrition Project	NR	24 mo.	Daily distribution of snacks to the school children based on the assigned group. Vegetarian supplement, milk supplement, meat supplement, and control (no food supplement provided).	Change in energy intake and markers of dietary quality among parents	Weak
Gunawardena et al., 2016 [28], Sri Lanka	CRC	Mothers of eighth-grade students: I: *n* = 152, C: *n* = 156	NR	NR (own experience)	12 mo.	Intervention group students were trained by facilitators through a series of discussions to acquire the ability to assess noncommunicable disease risk factors in their homes and take action to address them	Mothers’ weight, BMI, and self-reported consumption of food items	Strong
He et al., 2015 [20], He et al., 2016 [19] China	CRCT	Fifth-grade students: I: *n* = 141, C: *n* = 138; Adult family members: I: *n* = 278, C: *n* = 275	School-EduSalt	NR	3.5 mo.	Children in the intervention group were educated on the harmful effects of salt and how to reduce salt intake within the schools’ usual health education lessons. Children then relayed these salt reduction messages to their families	Salt intake (as measured by 24-h urinary sodium excretion), BP, and iodine consumption among adult family members	Strong
Katz et al., 2011 [29], USA	CRCT	Second- to fourth-grade students: I: *n* = 628, C: *n* = 552	The Nutrition Detectives program	Social-Ecological Model	NR	Five mini-lessons and family outreach	Dietary pattern of parents	Weak
Knight et al., 1991 [30], Jamaica	Non-RCT	Fourth- and fifth-grade students: I: *n* = 423, C: *n* = 199; Mothers/guardians: I: *n* = 90, C: *n* = 47	A child-to-child programme	NR	During the school year	Bi-weekly teacher training sessions to review what should be taught in the following two weeks and to assist in developing the curriculum. Action-oriented lessons for children.	Mothers’ nutritional knowledge	Weak
Newell et al., 2004 [31], Australia	Non-RCT	Third- to sixth-grade students: I: *n* = 307, C: *n* = 85; Parents: *n* = 613	The Tooty Fruity Vegie project	NR	2 yr.	Multi-strategy program including classroom-oriented strategies, parent-oriented strategies, school environment-oriented strategies, and school canteen-oriented strategies	Parental knowledge about recommended FV intakes	Weak
Øvrum and Bere 2014 [32], Norway	Natural experiment	First- to seventh-grade students; Parents: *n* = 1423	Norwegian School Fruit Scheme	NR	NR	Free daily fruit or vegetable or subscription to one fruit or vegetable per day at a subsidized price	Parents’ FV intake	Moderate
Perry et al., 1998 [33], USA	CRCT (matched pair)	Fourth-grade students; Parents: *n* = 324	5- a- day Power Plus	SLT	7 mo.	Behavioral curricula in the fourth and fifth grades, parental involvement/education, school foodservice changes, and industry involvement and support	Parents’ FV intake	Moderate
Reynolds et al., 2000 [34], USA	CRCT	Fourth-grade students; Parents: *n* = 1698	The High 5 Project	SCT	Fall/winter 1994/95	A classroom component with 14 lessons curriculum, in addition to booster sessions delivered during the second year; a parent component; and a food service component	Parents’ FV intake, knowledge of 5 a day and knowledge of low-fat food preparation	Strong
Sharma et al., 2016 [35], USA	Non-RCT	First-grade students. Parent-child dyads: I: *n* = 407, C: *n* = 310	Brighter Bites	SCT, TPB	16 wk.	Weekly distribution of fresh produce; nutrition education in schools and for parents; and weekly recipe tastings.	Parents’ FV intake	Moderate
Shi-Chang et al., 2004 [36], China	Non-RCT	Third- to fifth-grade primary students: *n* = 2575; First- and second-grade secondary students: *n* = 4277 (baseline survey); Parents and guardians: *n* = 998	Health- promoting schools	NR	18 mo.	School-wide health promotion activities, including school-based working groups; nutrition training for school staff; distribution of materials on school nutrition; nutrition education for students; student competitions; school-wide health promotion efforts; and outreach to families and communities	Nutrition knowledge of parents and guardians	Weak
Tak et al., 2007 [22] Tak et al., 2009 [21], Netherlands	Non-RCT.	Fourth-grade students: *n* = 953; Parents: *n* = 705 Fourth-grade students: I: *n* = 346, C: *n* = 425; Parents: I: *n* = 148 I, C: *n* = 287	‘Schoolgruiten’	NR	NR	Improvement of FV availability and accessibility, bi-weekly free FV distribution at the mid-morning break, and school curriculum aimed at increasing knowledge and skills related to FV consumption	Parental knowledge about recommendations for fruit	Weak
Te Velde et al., 2008 [37], Spain, Norway and the Netherlands	CRCT	Fifth- and sixth-grade students; Mothers or female guardians: I: *n* = 415, C: *n* = 838 (baseline survey)	Pro Children intervention	NR	NR	A classroom component, a school component, a family component, and one optional component, which differed slightly between intervention sites	Total intake of FV and the intake of FV separately among mothers or female guardians	Moderate
Wang et al., 2016 [38], China	CRCT	Parents of seventh-grade students: I: *n* = 62, C: *n* = 61	Health-promoting school	NR	6 mo.	Health-promoting school intervention consisting of a wide range of health promotion activities in different domains including, School environment, curriculum, and Family involvement	Parents’ weekly frequency of consumption of food items, and nutrition knowledge	Strong
Woodhouse et al., 2012 [39], UK	Non-RCT (pilot)	Primary school students; Parents: I: *n* = 47 intervention (baseline survey)	Bostin Value	NR	20 mo.	FV stall operated in the school playground twice a week, added initiatives (a loyalty card, 100 challenge week), family cooking sessions, and children’s tasting sessions	Parents’ FV consumption	Weak

Abbreviations: RCT = Randomized controlled trial, CRCT = Cluster randomized controlled trial; I = Intervention; C = Control; mo. = month; wk. = week; FV = Fruits and vegetables; BMI = Body mass index; SCT = Social Cognitive Theory; SLT = Social Learning Theory; TPB = Theory of Planned Behavior; NR = Not reported; BP = Blood pressure.

**Table 2:** Parental involvement, outcomes of interest, outcome measures, and findings on parental/family outcomes of interest.

Authors, Year	Parental Involvement	Outcomes of Interest	Outcome Measures	Findings
Bjelland et al., 2015, [23]	Fact sheets, brochures, and information sheets	Parental intakes of sugar-sweetened soft drinks and sugar-sweetened fruit drinks; Intake of FV	Self-administered questionnaire	Non-significant increase in maternal mean intake of fruits in the intervention group (mean = 9.1 servings/week) as compared to control group (mean = 8.4 servings/week) (*p* = 0.06).
Blom-Hoffman et al., 2008, [24]	Five interactive children’s books designed to complete with adult assistance to communicate a simple health message that the students learned at school	Knowledge of the “5 a Day” message	Parental interview using structured questionnaire	When compared to baseline, parents of the experimental group had higher percentage of correct answers about the “5 a Day” message at 1 year post baseline (21.6% higher, *p* < 0.05) and at 2 years post baseline (43.3% higher, *p* < 0.001). When compared to parent of the control group, they also had higher percentage of correct answer at 1 year post baseline (20.8% higher, *p* < 0.05), and at 2 years post baseline (40.1% higher, *p* < 0.001).
Burgess-Champoux et al., 2008, [25]	Weekly parent newsletters; bakery and grocery store tours; a ‘Whole Grain Day’ event	Self-reported intake of refined- and whole-grain foods	12-item food frequency section modified from the Block FFQ (Intake over the past month)	Self-reported intake of refined-grain foods decreased significantly for parents in the intervention school (pre-post difference: −0.3, *p* < 0.05) compared to those in comparison school (*p* < 0.01) post-intervention. No significant group differences were found in whole grain intake.
Crockett et al., 1989, [26]	Hearty Heart and Friends: children’s activity and eating records brought home for discussion, curriculum books, worksheets and children’s homework assignments brought home by the child; Hearty Heart Home Team: weekly mails received by child and parent team including a rule book with instructions about the week’s activities, a Hearty Heart adventure storybook, a scorecard, equipment, souvenirs, poster-size team tips, and incentives for completing activities.	Knowledge about diet and its relationship to cardiovascular disease	Self-administered questionnaire (Three scales)	Home Team alone group and the Hearty Heart and Home Team group had significantly greater knowledge scores as compared to the control group and Hearty Heart alone groups across all three scales.
		Dietary intake	Willett FFQ (Intake over the past two months)	There were no significant differences in dietary intake among the groups.
Dong et al., 2019, [18]	Four parent training meetings; 16 bi-weekly text messages about salt reduction.	Change in knowledge of salt intake	Self-administered questionnaire	After the intervention, intervention group parents had significantly higher awareness of the salt reduction knowledge than those of the control group (*p* < 0.01)
Gewa et al., 2013, [27]	/	Change in energy intake and markers of dietary quality among parents	Three non-consecutive 24 h recalls	A general decline in food quality and quantity was reported among parents of all groups. The decline in food quantity was only significant for the parents of the vegetarian group. Significant declines on at least one of the markers of dietary quality were seen among parents of vegetarian, meat, and control group.
Gunawardena et al., 2016, [28]	/	Mothers’ weight and BMI	Weight and height were measured by research team	Intervention group mothers had a significantly lower mean weight and BMI than as compared to control group (*p* < 0.0001); mean effect (95% CI) −2.49 (−3.38 to −1.60) kg for weight and −0.99 (−1.40 to −0.58) kg/m^2^ for BMI.
		Mothers’ self-reported consumption of FV, whole-grain product, pulse as main dish, deep fried foods and SSBs	27- item self-administered FFQ	No significant difference in individual-level food consumption between mothers of the two groups after the intervention.
He et al., 2015 [20], He et al., 2016 [19]	Educational materials in the form of a newsletter	Salt and iodine consumption among adult family members	24-h urinary sodium and iodine excretion	The mean effect (95% CI) on salt consumption for adults in the intervention group compared to control group was −2.9 g/day (−3.7 to −2.2 g; *p* < 0.001), representing a 25% reduction. The mean effect on iodine was −11.4% (*p* = 0.03).
		BP among adult family members	Trained researchers measured BP and pulse rate using a validated automatic blood pressure monitor	Compared to baseline, adults in both groups had an increase in systolic and diastolic BP. The increase in systolic BP was smaller in intervention group. The mean effect (95% CI) on systolic BP was −2.3 mmHg (−4.5 to −0.04 mmHg; *p* < 0.05). The effect on diastolic BP was not significant.
Katz et al., 2011, [29]	Written materials and/or parent information nights to introduce parent to the program	Dietary pattern of parents	Harvard Services FFQ	No significant improvements in dietary patterns from baseline between the parents of students in either group.
Knight et al. 1991, [30]	/	Mothers’ Nutritional knowledge	Interviewer- administered questionnaire	Mothers or guardians in the intervention group had significantly higher improvement in the mean nutrition score (from 4.17 to 5.10) compared to those from the control group (from 4.45 to 4.57) *p* < 0.05
Newell et al., 2004, [31]	Cooking classes, FV promoting flyers and newsletter articles, FV tasting, FV promoting merchandise, Competition asking parents to send in their handy hints for getting their children to eat FV.	Parents’ knowledge about recommended fruit and vegetable intakes	Self-administered survey	Significantly more intervention school parents as compared to control parents correctly identified recommended daily fruit intake of two servings (72% vs. 63%; continuity adjusted χ2 = 4.313, *p* < 0.05) and recommended daily vegetables intake of three servings (48% vs. 28%; continuity adjusted χ2= 17.062, *p* < 0.0001)
Øvrum and Bere 2014, [32]	/	Parents’ FV intake	Internet survey	Parents of children who received free fruit at school ate on average 0.19 more portions of fruits daily or 12.5% more fruits than parents of children who attend schools with no fruit arrangement (*p* = 0.04). No significant differences in vegetable intake between groups were found.
Perry et al., 1998, [33]	Fourth grade parental involvement: 5 information /activity packet brought home by students to be completed with parents; Fifth grade parental involvement: 4 snack packs, contained food items, that students brought home to prepare as a snack for their families.	Parents’ FV intake	Telephone survey (Single item measured average FV intake)	No significant differences in average fruit and vegetable consumption between groups were observed.
Reynolds et al., 2000, [34]	Program overview at parent kick off night, assignment to be completed with children, brochures, and skills building materials	Parents’ FV intake,	Self-administered questionnaire (FV items from Health Habits and History Q)	Post-intervention: intervention group parents consumed more servings of FV combined compared to control parents (+0.29, *p* < 0.04); When examined separately, the difference between conditions was significant for vegetables (+0.17, *p* < 0.04) but not for fruit consumption; 1-year post-intervention: No differences were observed for parental FV consumption.
		Parental knowledge of 5 a day & knowledge of low-fat food preparation	Self-administered questionnaire	Post-intervention: a significant positive effect on knowledge of five a day serving among intervention group. No significant differences on knowledge of low-fat preparation among intervention and control groups. 1-years post intervention: no significant differences on nutrition knowledge between groups
Sharma et al., 2016, [35]	Weekly distribution of produce and healthy recipe tastings during pick up time, health education in schools and for parents, handbooks and weekly recipe cards sent home with the parents.	Parents’ FV intake	The validated 10-item FV Screener by the National Institutes of Health (Intake over the past month)	Intervention group parents had a significant increase in fruit consumption from baseline to midpoint (8 weeks follow up) (+25 servings-day, *p* = 0.03) and post intervention (16 weeks follow up) (+0.25 servings-day, *p* = 0.01) compared to those in the control group. A significant increase in vegetable (+0.30 servings/day, *p* = 0.04) and in total FV consumption (+53 serving/day, *p* = 0.007) was also seen among parents of the intervention groups compared to those in the control group at midpoint assessment, but not post-intervention.
Shi-Chang et al., 2004, [36]	Students passing information about nutrition to their families, parents’ leaflet on healthy nutrition and school lunch menus that they could prepare at home, lectures and workshops at schools.	Nutrition knowledge	Questionnaires	Parents and guardians of the pilot schools demonstrated higher knowledge gain than those of the control schools in three areas: nutrients and their functions, Chinese dietary guidelines and adequate dietary principles. They also increased their knowledge in the areas of nutritional deficiencies and their symptoms (from 35% to 66.2%, *p* < 0.01) and nutrient-rich foods (from 38.8% to 66.8%, *p* < 0.01), while knowledge of these areas did not change significantly amongst parents and guardians at control schools.
Tak et al., 2007, [22] Tak et al., 2009, [21]	/	Knowledge of parent about recommendations for fruit	Self-administered survey	No significant differences were observed between groups at the first- and the second- year follow-ups.
Te Velde et al., 2008, [37]	Parents were encouraged to be involved in the project through their children’s homework assignments, parental newsletters and a parent version of the web-based computer-tailored tool for personalized feedback on their intake	Total intake of FV and the intake of FV separately among mothers or female guardians	24-h recall	No significant intervention effects were observed regarding FV intake of the mothers at the first- and second-year follow-ups.
Wang et al., 2016, [38]	One 90-min lecture, distribution of publicity resources for parents (one time, before the workshop), monthly short message to parents	Frequency of consumption of food items among parents	FFQ (intake over the past seven days)	No significant differences were observed in parents’ consumption of different food items between the HPS School and the control school
		Parents’ nutrition knowledge	Self-administered questionnaire	Significant difference in parents’ awareness rate of eight knowledge items (out of ten) between the HPS School and the Control School after intervention (*p* < 0.05)
Woodhouse et al., 2012, [39]	FV stall operated in the school playground twice a week, recipe cards, loyalty cards, family cooking sessions, and different promotion activities to promote take up for the “Family Cooking Sessions”	Parents’ FV intake	Self-administered survey	At the first follow-up, the mean portions of FV consumed by parents of the pilot school increased significantly from 2.4 to 3.1 (*p* = 0.03) for fruits and from 2.7 to 3.4 (*p ≤* 0.001) for vegetables. The increase in fruits consumption was significantly higher than that of the comparison school (*p* = 0.02); At the second follow-up, the average of FV consumption by parents/carers at pilot school was not significantly different from either the baseline or the comparison school.

Abbreviations: B = Blood pressure; FFQ = Food frequency questionnaire; FV = Fruits and vegetables; BMI = Body mass index; SSB = Sugar sweetened beverages; HPS = Health promoting schools; / = No parental involvement.

## Data Availability

All literature reviewed in the study was publicly available.

## Supplementary Materials

The following supporting information can be downloaded at: https://www.mdpi.com/article/10.3390/nu14122399/s1, Table S1: Full search strategy for all databases, Table S2: Quality assess- ment of included studies using the EPHPP tool, Table S3: PRISMA 2020 Checklist, Table S4: Summary table of the impact of school-based nutrition interventions on parental outcomes, Figure S1: Summary of school-based nutrition intervention impacts on parental outcomes.
