# Supplementary material for: The Impact of School-Based Nutrition Interventions on Parents and Other Family Members: A Systematic Literature Review
Source: Nutrients. 2022 Jun 9;14(12):2399. doi: 10.3390/nu14122399 (PMC9231235; doi:10.3390/nu14122399)
Supplement: Supplementary file 1 [file nutrients-14-02399-s001.zip › E.A.Supplematary materials -final.pdf]

SUPPLEMENTARY MATERIALS

Table S1. Search strategy for all databases………………...…………………………2-3

Table S2. Summary table of the quality assessment using the EPHPP tool……….….4

Table S3. PRISMA 2020 Checklist…………………...……………………………...5-8

Table S4. Summary table of the impact of school-based nutrition interventions on parental outcomes…………………………………………………….…..…9-10

Figure S1. Summary of school-based nutrition intervention impacts on parental outcomes…………………………………….….……………………………...10

Tables S1. Search strategy for different databases

PubMed

Search terms related to setting	Search terms related to intervention	Search terms related to target population
	AND	AND
("Schools"[Mesh]OR primary school* OR secondary school* OR “School based” OR “school centered” OR “school centred”)	"Health Education"[Mesh] OR “health education”	parent OR father OR mother OR caregiver OR family
("Schools"[Mesh]OR primary school* OR secondary school* OR “School based” OR “school centered” OR “school centred”)	“Intervention “	parent OR father OR mother OR caregiver OR family
("Schools"[Mesh]OR primary school* OR secondary school* OR “School based “OR “school centered” OR “school centred”)	Nutrition education OR "Diet, Food, and Nutrition/education"[Mesh]	parent OR father OR mother OR caregiver OR family

Web of Science

Search terms related to setting	Search terms related to intervention	Search terms related to target population
	AND	AND
(primary school* OR secondary school* OR “School based” OR “school centered” OR “school centred”)	(Health OR nutrition) NEAR/5 Education	parent* OR father* OR mother* OR caregiver* OR famil*)
(primary school* OR secondary school* OR “School based” OR “school centered” OR “school centred”)	(Health OR nutrition) NEAR/5 Intervention	parent* OR father* OR mother* OR caregiver* OR famil*)
(primary school* OR secondary school* OR “School based” OR “school centered” OR “school centred”)	(Health OR nutrition) NEAR/5 promotion	parent* OR father* OR mother* OR caregiver* OR famil*)

PsycINFO and EconLit. through EBESCO

Search terms related to setting	Search terms related to intervention	Search terms related to target population
	AND	AND
(primary school* OR secondary school* OR “School based “OR “school centered” OR “school centred”)	(Health Education OR nutrition Education)	parent* OR father* OR mother* OR caregiver* OR famil*
(primary school* OR secondary school* OR “School based “OR “school centered” OR “school centred”)	(Health Intervention OR nutrition Intervention)	parent* OR father* OR mother* OR caregiver* OR famil*
(primary school* OR secondary school* OR “School based “OR “school centered” OR “school centred”)	(Health promotion OR nutrition promotion)	parent* OR father* OR mother* OR caregiver* OR famil*

Cochrane reviews

Search terms related to setting	Search terms related to intervention	Search terms related to target population
	AND	AND
(primary school* OR secondary school* OR “School based” OR “school centered” OR “school centred”)	(Health Education OR nutrition Education)	parent* OR father* OR mother* OR caregiver* OR famil
(primary school* OR secondary school* OR “School based” OR “school centered” OR “school centred”)	(Health Intervention OR nutrition Intervention)	parent* OR father* OR mother* OR caregiver* OR famil
(primary school* OR secondary school* OR “School based” OR “school centered” OR “school centred”)	(Health promotion OR nutrition promotion)	parent* OR father* OR mother* OR caregiver* OR famil

Google Scholar

Search terms related to setting	Search terms related to intervention	Search terms related to target population
	AND	AND
(primary school* OR secondary school* OR “School based” OR “school centered” OR “school centred”)	(Health Education OR nutrition Education)	parent* OR father* OR mother* OR caregiver* OR famil*)
(primary school* OR secondary school* OR “School based” OR “school centered” OR “school centred”)	(Health Intervention OR nutrition Intervention)	parent* OR father* OR mother* OR caregiver* OR famil*)
(primary school* OR secondary school* OR “School based” OR “school centered” OR “school centred”)	(Health promotion OR nutrition promotion)	parent* OR father* OR mother* OR caregiver* OR famil*)

Table S2. Summary table of the quality assessment using the EPHPP tool

Author, Year	Selection Bias	Study Design	Confounders	Blinding	Data Collection Methods	Withdrawals & Dropouts	Global Rating
(Bjelland et al. 2015)	Moderate	Strong	Moderate	Moderate	Weak	Weak	Weak
(Blom-Hoffman et al. 2008)	Weak	Weak	Weak	Moderate	Weak	Weak	Weak
(Burgess-Champoux et al. 2008)	Weak	Strong	Moderate	Moderate	Moderate	Moderate	Moderate
(Crockett et al. 1989)	Moderate	Strong	Strong	Moderate	Strong	Moderate	Strong
(Dong et al. 2019)	Moderate	Strong	weak	Moderate	Weak	Strong	Weak
(Gewa et al. 2013)	Moderate	Strong	Strong	Weak	Weak	Strong	Weak
(Gunawardena et al.2016)	Strong	Strong	Strong	Moderate	Moderate	Strong	Strong
(He et al. 2015)	Moderate	Strong	Strong	Moderate	Strong	Strong	Strong
(He et al. 2016)	Moderate	Strong	Strong	Moderate	Strong	Strong	Strong
(Katz et al.2011)	Moderate	Strong	Weak	Moderate	Strong	Weak	Weak
(Knight et al.1991)	Moderate	Weak	Strong	Moderate	Weak	Strong	Weak
(Newell et al.2004)	Weak	Strong	weak	Moderate	Moderate	Weak	Weak
(Øvrum and Bere 2014)	Moderate	Moderate	Strong	Moderate	Weak	Moderate	Moderate
(Perry et al. 1998)	Moderate	Strong	Strong	Moderate	Weak	Strong	Moderate
(Reynolds et al. 2000)	Moderate	Strong	strong	Moderate	Strong	Moderate	Strong
(Sharma et al.2016)	Weak	Strong	Strong	Moderate	Moderate	Moderate	Moderate
(Shi-Chang et al. 2004)	Moderate	Weak	Weak	Moderate	Moderate	Weak	Weak
(Tak et al. 2007)	Strong	Strong	Strong	Moderate	Weak	Weak	Weak
(Tak et al. 2009)	Strong	Strong	Strong	Moderate	Weak	Weak	Weak
(Te Velde et al. 2008)	Moderate	Strong	Strong	Moderate	Strong	Weak	Moderate
(Wang et al. 2016)	Moderate	Strong	Strong	Moderate	Strong	Strong	Strong
(Woodhouse et al. 2012)	Weak	Weak	Weak	Moderate	Weak	Weak	Weak

Table S3. PRISMA 2020 Checklist

Section and Topic	Item #	Checklist item	Location where item is reported
**TITLE**			
Title	1	Identify the report as a systematic review.	Title page (Page1)
**ABSTRACT**			
Abstract	2	See the PRISMA 2020 for Abstracts checklist.	Table S3 (see below)
**INTRODUCTION**			
Rationale	3	Describe the rationale for the review in the context of existing knowledge.	Page 1-2
Objectives	4	Provide an explicit statement of the objective(s) or question(s) the review addresses.	Page 2
**METHODS**			
Eligibility criteria	5	Specify the inclusion and exclusion criteria for the review and how studies were grouped for the syntheses.	Page 2
Information sources	6	Specify all databases, registers, websites, organisations, reference lists and other sources searched or consulted to identify studies. Specify the date when each source was last searched or consulted.	Page 2
Search strategy	7	Present the full search strategies for all databases, registers and websites, including any filters and limits used.	Tables S1
Selection process	8	Specify the methods used to decide whether a study met the inclusion criteria of the review, including how many reviewers screened each record and each report retrieved, whether they worked independently, and if applicable, details of automation tools used in the process.	Page 3
Data collection process	9	Specify the methods used to collect data from reports, including how many reviewers collected data from each report, whether they worked independently, any processes for obtaining or confirming data from study investigators, and if applicable, details of automation tools used in the process.	X
Data items	10a	List and define all outcomes for which data were sought. Specify whether all results that were compatible with each outcome domain in each study were sought (e.g. for all measures, time points, analyses), and if not, the methods used to decide which results to collect.	Page 2
	10b	List and define all other variables for which data were sought (e.g. participant and intervention characteristics, funding sources). Describe any assumptions made about any missing or unclear information.	Page 3
Study risk of bias assessment	11	Specify the methods used to assess risk of bias in the included studies, including details of the tool(s) used, how many reviewers assessed each study and whether they worked independently, and if applicable, details of automation tools used in the process.	Page 3
Effect measures	12	Specify for each outcome the effect measure(s) (e.g. risk ratio, mean difference) used in the synthesis or presentation of results.	X
Synthesis methods	13a	Describe the processes used to decide which studies were eligible for each synthesis (e.g. tabulating the study intervention characteristics and comparing against the planned groups for each synthesis (item #5)).	X
	13b	Describe any methods required to prepare the data for presentation or synthesis, such as handling of missing summary statistics, or data conversions.	X
	13c	Describe any methods used to tabulate or visually display results of individual studies and syntheses.	X
	13d	Describe any methods used to synthesize results and provide a rationale for the choice(s). If meta-analysis was performed, describe the model(s), method(s) to identify the presence and extent of statistical heterogeneity, and software package(s) used.	X
	13e	Describe any methods used to explore possible causes of heterogeneity among study results (e.g. subgroup analysis, meta-regression).	X
	13f	Describe any sensitivity analyses conducted to assess robustness of the synthesized results.	X
Reporting bias	14	Describe any methods used to assess risk of bias due to missing results in a synthesis (arising from reporting biases).	X

Table S3. PRISMA 2020 Checklist

Section and Topic	Item #	Checklist item	Location where item is reported
assessment			
Certainty assessment	15	Describe any methods used to assess certainty (or confidence) in the body of evidence for an outcome.	X
**RESULTS**			
Study selection	16a	Describe the results of the search and selection process, from the number of records identified in the search to the number of studies included in the review, ideally using a flow diagram.	Page 3, Figure 1
	16b	Cite studies that might appear to meet the inclusion criteria, but which were excluded, and explain why they were excluded.	X
Study characteristics	17	Cite each included study and present its characteristics.	Table 1, Page 8,
Risk of bias in studies	18	Present assessments of risk of bias for each included study.	Page 8, Table S2
Results of individual studies	19	For all outcomes, present, for each study: (a) summary statistics for each group (where appropriate) and (b) an effect estimate and its precision (e.g. confidence/credible interval), ideally using structured tables or plots.	X
Results of syntheses	20a	For each synthesis, briefly summarise the characteristics and risk of bias among contributing studies.	X
	20b	Present results of all statistical syntheses conducted. If meta-analysis was done, present for each the summary estimate and its precision (e.g. confidence/credible interval) and measures of statistical heterogeneity. If comparing groups, describe the direction of the effect.	X
	20c	Present results of all investigations of possible causes of heterogeneity among study results.	X
	20d	Present results of all sensitivity analyses conducted to assess the robustness of the synthesized results.	X
Reporting biases	21	Present assessments of risk of bias due to missing results (arising from reporting biases) for each synthesis assessed.	X
Certainty of evidence	22	Present assessments of certainty (or confidence) in the body of evidence for each outcome assessed.	X
**DISCUSSION**			
Discussion	23a	Provide a general interpretation of the results in the context of other evidence.	Page 14-15
	23b	Discuss any limitations of the evidence included in the review.	Page 15-16
	23c	Discuss any limitations of the review processes used.	X
	23d	Discuss implications of the results for practice, policy, and future research.	Page 15
**OTHER INFORMATION**			
Registration and protocol	24a	Provide registration information for the review, including register name and registration number, or state that the review was not registered.	Page 2
	24b	Indicate where the review protocol can be accessed, or state that a protocol was not prepared.	X
	24c	Describe and explain any amendments to information provided at registration or in the protocol.	X
Support	25	Describe sources of financial or non-financial support for the review, and the role of the funders or sponsors in the review.	Page 16
Competing interests	26	Declare any competing interests of review authors.	Page 16

Table S3. PRISMA 2020 Checklist

Section and Topic	Item #	Checklist item	Location where item is reported
Availability of data, code and other materials	27	Report which of the following are publicly available and where they can be found: template data collection forms; data extracted from included studies; data used for all analyses; analytic code; any other materials used in the review.	Page 16

From: Page MJ, McKenzie JE, Bossuyt PM, Boutron I, Hoffmann TC, Mulrow CD, et al. The PRISMA 2020 statement: an updated guideline for reporting systematic reviews. BMJ 2021;372:n71. doi: 10.1136/bmj.n71

For more information, visit: http://www.prisma-statement.org/

PRISMA 2020 for Abstracts Checklist

Section and Topic	Item #	Checklist item	Reported (Yes/No)
**TITLE**			
Title	1	Identify the report as a systematic review.	Yes
**BACKGROUND**			
Objectives	2	Provide an explicit statement of the main objective(s) or question(s) the review addresses.	Yes
**METHODS**			
Eligibility criteria	3	Specify the inclusion and exclusion criteria for the review.	Yes
Information sources	4	Specify the information sources (e.g. databases, registers) used to identify studies and the date when each was last searched.	Yes
Risk of bias	5	Specify the methods used to assess risk of bias in the included studies.	No
Synthesis of results	6	Specify the methods used to present and synthesise results.	No
**RESULTS**			
Included studies	7	Give the total number of included studies and participants and summarise relevant characteristics of studies.	Yes
Synthesis of results	8	Present results for main outcomes, preferably indicating the number of included studies and participants for each. If meta-analysis was done, report the summary estimate and confidence/credible interval. If comparing groups, indicate the direction of the effect (i.e. which group is favoured).	Yes
**DISCUSSION**			
Limitations of evidence	9	Provide a brief summary of the limitations of the evidence included in the review (e.g. study risk of bias, inconsistency and imprecision).	No
Interpretation	10	Provide a general interpretation of the results and important implications.	Yes
**OTHER**			
Funding	11	Specify the primary source of funding for the review.	No
Registration	12	Provide the register name and registration number.	No

From: Page MJ, McKenzie JE, Bossuyt PM, Boutron I, Hoffmann TC, Mulrow CD, et al. The PRISMA 2020 statement: an updated guideline for reporting systematic reviews. BMJ 2021;372:n71. doi: 10.1136/bmj.n71

For more information, visit: http://www.prisma-statement.org/

Table S4. Summary table of the impact of school-based nutrition interventions on parental outcomes

Outcomes of interest	Follow-up period	Impact
**Nutrition knowledge**		
Knowledge of the "5 a Day" message [24]	1-year post baseline	+
Knowledge of the "5 a Day" message [24]	2-years post baseline	+
Parental knowledge of 5 a day servings [34]	Post- intervention	+
Parental knowledge of 5 a day servings [34]	1-year post intervention	-/+
Parental knowledge of low-fat food preparation [34]	Post- intervention	-/+
Parental knowledge of low-fat food preparation [34]	1-year post intervention	-/+
Parents' knowledge about recommended fruit and vegetable intakes [31]	Post- intervention	+
Knowledge of parent about recommendations for fruit intake [21], [22]	First-year follow-up	-/+
Knowledge of parent about recommendations for fruit intake [21], [22]	Second-year follow-up	-/+
Knowledge about diet and its relationship to cardiovascular disease [26]	Post- intervention	+
Change in knowledge of salt intake [18]	Post- intervention	+
Mothers' Nutritional knowledge [30]	Post-intervention	+
Nutrition knowledge [36]	Post- intervention	+
Parents' nutrition knowledge [38]	Post- intervention	-/+
**Dietary intake**		
Self-reported intake of refined- grain food [25]	Post- intervention	+
Self-reported intake of whole-grain foods [25]	Post- intervention	-/+
Mothers' self-reported consumption of FV, whole-grain product, pulse as main dish, deep fried foods and SSBs [28]	Post- intervention	-/+
Parental intakes of sugar-sweetened soft drinks and sugar-sweetened fruit drinks; Intake of FV [23]	Post- intervention	-/+
Parents' fruits intake [32]	During intervention (Natural experiment)	+
Parents' vegetables intake [32]	During intervention (Natural experiment)	-/+
Parents' FV intake [33]	Post- intervention	-/+
Parents' fruits intake [34]	Post-intervention	-/+
Parents' vegetables intake [34]	Post-intervention	-/+
Parents' FV intake [34]	1-year post-intervention	-/+
Parents' fruits intake [35]	8 weeks post-baseline	+
Parents' fruits intake [35]	Post intervention	+
Parents' vegetables intake [35]	8 weeks post-baseline	+
Parents' vegetables intake [35]	Post intervention	-/+
Total intake of FV and the intake of FV separately among mothers or female guardians [37]	post- intervention	-/+
Total intake of FV and the intake of FV separately among mothers or female guardians [37]	1-year post- intervention	-/+
Parents' FV intake [39]	15 months post-baseline	+
Parents' FV intake [39]	Post-intervention	-/+
Salt consumption among adult family members [20]	Post- intervention	+
Frequency of consumption of food items among parents [38]	Post-intervention	-/+

Dietary intake [26]	Post- intervention	-/+
Dietary pattern of parents [29]	Post- intervention	-/+
Change in energy intake and markers of dietary quality among parents [27]	Post- intervention	-
**Health outcomes**		
Mothers' weight and BMI [28]	Post-intervention	+
BP among adult family members [20], [19]	Post- intervention	+

Abbreviations: (-/+) = No change; (+) = Positive change; (-) = Negative change; FV = Fruits and vegetables; SSBs = Sugar sweetened beverages; BMI= Body mass index; BP: Blood pressure

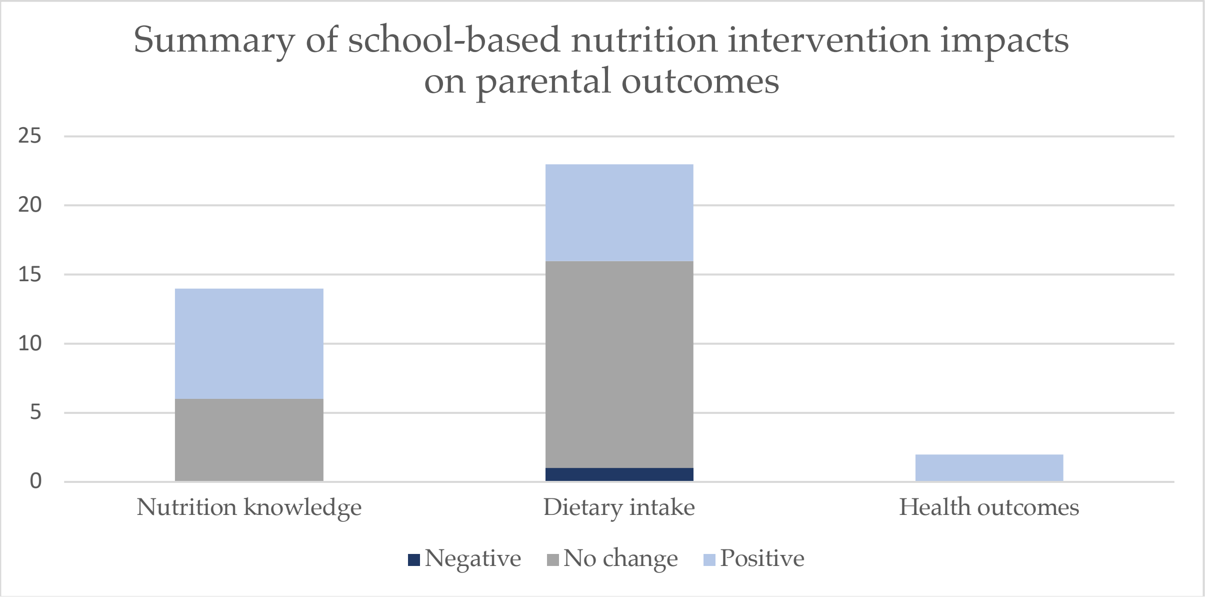

Figure S1. Summary of school-based nutrition intervention impacts on parental outcomes.
Note: Nutrition knowledge: 14 outcomes based on 10 studies; Dietary intake: 23 outcomes based on 14 studies; Health outcomes: 2 outcomes based on 2 studies
